# Efficient Removal of Pb(II) from Aqueous Solutions by Using Oil Palm Bio-Waste/MWCNTs Reinforced PVA Hydrogel Composites: Kinetic, Isotherm and Thermodynamic Modeling

**DOI:** 10.3390/polym12020430

**Published:** 2020-02-12

**Authors:** Muhammad Zulfiqar, San Yi Lee, Amira Azreena Mafize, Nur Adlin Mastura Abdul Kahar, Khairiraihanna Johari, Nurul Ekmi Rabat

**Affiliations:** Chemical Engineering Department, Universiti Teknologi PETRONAS, Seri Iskandar 32610, Perak Darul Ridzuan, Malaysia; zulfiqar_rehmani@hotmail.com (M.Z.); 8lsy95@gmail.com (S.Y.L.); amiraazreena7550@gmail.com (A.A.M.); adlinmasturakahar@gmail.com (N.A.M.A.K.); khairiraihana.j@utp.edu.my (K.J.)

**Keywords:** hydrogel, polyvinyl alcohol, adsorption, multiwalled carbon nanotubes, oil palm empty fruit bunch, mechanism

## Abstract

Polyvinyl alcohol (PVA) hydrogel are still restricted for some applications because their lower mechanical strength and thermal stability. The PVA-based composites are drawing attention for the removal of heavy metals based on their specific functionality in adsorption process. The main objective of this work is to synthesize oil palm bio-waste (OPB)/multiwalled carbon nanotubes (MWCNTs) reinforced PVA hydrogels in the presence of *N*,*N*′-methylenebisacrylamide (NMBA) as a crosslinking agent and ammonium persulfate (APS) as an initiator via simple in-situ polymerization technique. The as-prepared reinforced nanocomposites were characterized by FESEM, BET surface area, differential scanning calorimetry (DSC), TGA and FTIR analysis. The possible influence of OPB and MWCNTs on the tensile strength, elongation at break and elastic modulus of the samples were investigated. It was found that reinforced nanocomposites exhibited enhanced mechanical properties as compared to non-reinforced material. The evaluation of reinforced nanocomposites was tested by the removal of Pb(II) aqueous solutions in a batch adsorption system. The pseudo-second-order kinetic model was used to illustrate the adsorption kinetic results and Langmuir isotherm was more suitable to fit the equilibrium results providing maximum adsorption capacities. The evaluation of thermodynamic parameters describes the spontaneous, endothermic and chemisorption adsorption process while activation energy reveals the physical adsorption mechanism. Therefore, the coordination effects among OPB, MWCNTs and PVA polymer hydrogels can produce a promising adsorbent material for wastewater treatment applications.

## 1. Introduction

Currently, wastewater purification techniques have attained much attention in environmental science because of water sources contamination from different toxic pollutants such as heavy metals, which cause several environmental, ecological and health problems [1,2,3]. The contaminants produce harmful effects on living organisms due to non-biodegradable properties and high toxicity even at low concentration [4]. Toxic heavy metals that contain wastewater greatly decrease microbial activity, thus adversely influence via biological treatment methods. Moreover, biological treatment is a complex process because of physicochemical, biological, operating and design conditions [5]. The release of industrial effluents into the open environment without any treatment has become a challenging topic and the control of water pollution has gained great importance in recent years. Therefore, it is necessary to treat such harmful compounds from wastewater before being released into the open atmosphere. Various treatment methods, such as chemical precipitation [6], adsorption [7], evaporation [8], reverse osmosis [9], ion exchange [10], membrane filtration [11] and biosorption [12] have been adopted for heavy metal ions elimination from different aqueous solutions. Among these, adsorption is considered as one of the best alternative processes to remove the heavy metal ions from wastewater by using simple, cheap and abundantly available adsorbents [4,12]. It has great potential to minimize biological and/or chemical sludge and able to detoxify the highly polluted effluents without using additional nutrients [12]. While remaining adsorption methods have some operational problems for example membrane filtration method require high energy and not suitable for large quantities of contaminated effluents. Similarly, chemical precipitation method also involved treatment of harmful sludge and disposal issues [13].

In recent years, hydrogels have received great interest from the scientific community and industrial sector due to their excellent characteristics, such as permeability, biocompatibilities, hydrophilic properties and high swelling ratio [14,15]. Hydrogels prepared from natural polymer especially polyvinyl alcohol (PVA) exhibited superior characteristics as compared to synthetic polymer-based hydrogels. PVA hydrogel is a semi-crystalline polymer synthesized from the hydrolysis of polyvinyl acetate and has good solubility, biocompatibility, biodegradability properties [16]. It has emerged as a potential adsorbent because of its high swelling capacity. It has strong ability to absorb a large amount of water molecules due to the electrostatic repulsion between the hydrophilic functional groups, which are connected to its polymeric strength and is the basis for the enlargement of the hydrogel connection system. Meanwhile, their resistance to dissolution is mainly due to the formation of cross-links between network chains [17]. As an adsorbent, the contaminants were captured between fine pores of hydrogel developed via crosslinking networks; hence, adsorbed [18]. The high-water-content and porous structure networks help to diffuse the solute through PVA hydrogel structure to trap the contaminants from wastewaters. Despite being commercially used in many applications, poor thermal stability, low solubility, non-biodegradability and poor mechanical strength are the main limitation of the PVA hydrogel [19]. Therefore, the formation of high-performance hydrogel composites is of great interest to improve its weak structure as well as its adsorption capacity. To solve these problems, some research studies have employed different types of fillers to acquire an encouraging breakthrough. 

Recently, interest has been focused to develop economical, green and safe adsorbents by using various bio-waste fillers as unique supporting materials for wastewater treatment. Chitosan is most widely used to improve the strength of hydrogel composites and has showed excellent adsorption capacity for Pb(II) from wastewater due to the rich carbonyl, amino and hydroxyl groups [20]. It is a well-known biopolymer, which is derived from alkaline N-deacetylation of chitin [21]. The development has been focused to prepare chitosan hydrogel beads, which are used for adsorption applications. Major limitations of chitosan hydrogel beads are poor mechanical and thermal properties as well as chemical resistance [22]. To improve these properties, epichlorohydrin, glutaraldehyde and ethylene glycol glycidyl ether have been commonly employed for the cross-linking of chitosan hydrogel beads and showed better thermal and mechanical behavior [22,23]. Unfortunately, cross-linking reduces the adsorption performance of chitosan. Various bio materials such as chitosan, alginate, cellulose and cell bio mass have been commonly employed for the development of innovative adsorbents and tested for environmental applications [3,24]. In recent years, chitosan has been incorporated with PVA and showed excellent adsorption, thermal and mechanical characteristics. Chitosan/PVA beads hydrogel composites have stated an adsorption capacity of 9.48 mg/g for Pb(II) [25]. This lower adsorption activity was due to the shorter contact time and lower Pb(II) concentration. Chitosan/PVA blend nanofiber membrane composites have successfully removed Pb(II) ions with maximum adsorption capacity of 266.12 mg/g [26]. Cellulose/chitosan/PVA nanofibrous films are proven to be more effective for the adsorption of Pb(II) ions with an adsorption capacity of 323.49 mg/g [20]. Chitosan/PVA talc composites have described the removal efficiency of 88 % for Pb(II) [27]. The addition of nanomaterials may also enhance the adsorption, mechanical and thermal characteristics of bio-waste-based PVA hydrogel composites due to the large specific surface area. Chitosan/multiwalled carbon nanotubes (MWCNTs)/PVA membrane composites showed excellent adsorption capacity for Pb(II) [28]. Chitosan/PVA thin membrane composites were prepared in spherical nano-diamonds particles and reported an adsorption capacity of 121.3 mg/g for Pb(II) [29]. Xanthate-modified with magnetite (Fe_3_O_4_)-based chitosan/PVA hydrogel showed 97.8% of adsorption efficiency for Pb(II) [30]. Although chitosan-based adsorbents have great attractions in adsorption application, they have some limitations, such as slow adsorption rate, swelling and minimized adsorption performance after several cycles [31]. The cross-linked α-manganese dioxide nanoparticles/PVA composites is used to increase the adsorption ability of Pb(II) to 88% [32]. MWCNTs/PVA hydrogel composites have exhibited superior removal efficiency of 86% for Pb(II) ions [33]. Fe_3_O_4_/PVA/spent coffee ground composites have successfully adsorbed Pb(II) ions with experimental adsorption capacity of 0.275 mmol/g [34]. PVA/graphene oxide-sodium alginate nanocomposite hydrogels have adsorption capacity of 279.43 mg/g for Pb(II) ions [35]. Pineapple peel/PVA hydrogel was successfully used for the preparation of nano-composites and used for the immobilization of papain with 79% activity [36]. Various PVA hydrogel-based nano-composites loaded with their different types of fillers along their adsorption capacities for Pb(II) are listed in Table 1. All these modifications can appraise the thermal behavior, mechanical stability, adsorption and desorption performance. Based on literature, a novel, economical and eco-friendly oil palm bio-waste (OPB) waste material for PVA hydrogel-based adsorbent is introduced.

Oil palm trees are agricultural plants and play an important role in the economic growth of Malaysia. OPB is biomass waste that is produced after palm oil is removed from the palm fruit for oil production [18,19]. It was found that the addition of OPB could greatly enhance the polymer hydrogel durability via increasing the fiber medium adherence into the hydrogel by granting an irregular surface. However, based on a previous research in [19], the addition of more than 15 wt % of OPB created a negative influence over the swelling behavior of hydrogel. The fine solid particles of OPB can provide a bigger specific surface area to build a possible approach and crosslinking with co-polymer, initiating the increment of crosslink density into the hydrogel. This high crosslink density subsequently limits the flexibility of hydrogel and produces a rigid structure, which is not able to expand for larger water absorbency. Therefore, low mechanical strength and thermal properties have limited the performance to a great extent. To improve these properties, different nanomaterials especially MWCNTs is added into the PVA hydrogel along with OPB and acts as the reinforcing agents. MWCNTs are a highly promising adsorbent type nanoparticle used for the modification of polymers in engineering and science for environmental applications [14]. They are known to be a highly efficient nanofiller by means of high porosity, low density, specific surface area, mechanical characteristics especially toughness with hollow and layered structures [15,37]. However, MWCNTs have a tendency to form agglomerate due to the Van der Waals forces and difficult to disperse and align in the polymer matrix, representing the main disadvantages [33,38]. It has been reported that MWCNTs have strong ability to remove the organic pollutants from wastewater owing to larger specific area, good thermal and mechanical characteristics [39]. These properties could provide useful information about the dispersion of MWCNTs into the polymer as well as interfacial interaction between filler and matrix. The mechanical properties are related to physical parameters such as porosity and cross-linking degree of the network. Therefore, to develop a MWCNTs-based nanocomposite, which can show great ability in enhancing the interfacial attraction and progress load is still not clear. The incorporation of OPB into PVA composites with MWCNTs has great potential to improve the thermal and mechanical properties, which make an attractive adsorbent for wastewater treatment. Therefore, based on literature, we are introducing for the first time, a novel, economical and eco-friendly OPB waste material for PVA hydrogel-based adsorbents along MWCNTs, which describe the clear novelty of present work. As a low cost with easy availability, oil palm bio-waste is an attractive and inexpensive option for the removal of heavy metal from wastewater. Furthermore, the formation of PVA hydrogels with the coordination of OPB and MWCNTs to study the different parameters including melting temperature, tensile strength, elongation at break and elastic modulus is not clear, which also shows the significance of present research.

In this work, high strength PVA hydrogels were successfully reinforced with OPB and MWCNTs by in-situ polymerization in the presence of NMBA as a crosslinking agent and APS as an initiator. The different influencing parameters namely OPB loading, solution pH, contact time, Pb^2+^ concentration and temperature were investigated by using PVA, OPB/PVA and OPB/PVA/MWCNTs hydrogel adsorbents. The as-prepared OPB/MWCNTs/PVA hydrogels composites have exhibited significant adsorption performance due to the large number of active sites for the adsorption of Pb(II) ions from wastewater. Moreover, kinetic, isotherm and thermodynamics were also studied to understand the adsorption mechanism of the newly developed composites. The reinforced and non-reinforced PVA hydrogels were characterized by using FESEM, BET surface area, TGA, differential scanning calorimetry (DSC) and FTIR analysis.

## 2. Materials and Methods 

### 2.1. Materials and Regents

The fully hydrolyzed PVA with an average molecular weight (*M*_w_) of 60,000 g/mol, *N*,*N*′-Methylenebisacrylamide (NMBA), ammonium persulfate (APS) and NaOH were supplied by Merck Sdn. Bhd., Selangor, Malaysia and used as received. The multi-walled carbon nanotubes (MWCNTs) with a diameter of 5–20 nm and specific surface area (BET) of 100–700 m^2^/g was purchased from Carbon Nano-Material Technology Co., Ltd, Gwangju, Korea. Oil palm bio-waste was obtained from Felda Nasaruddin Oil Palm Plantation, Selangor, Malaysia. The standard stock solutions of Pb(II) from Pb(NO_3_)_2_ were initially prepared. 

### 2.2. Pretreatment of OPB Fiber

The raw OPB fibers were obtained from the fruit pulps and the moisture was removed in sunlight. The dried fiber was soaked with NaOH and hot water at 80 °C for 6 h. The treated OPB was oven dried at 70 °C for 24 h. Then, as-obtained fiber was ground and sieved into 100-micrometer size using a sieve shaker (Endecotts, MINOR-200, London, UK).

### 2.3. Preparation of OPB/MWCNTs Reinforced PVA Hydrogel

The OPB/MWCNTs reinforced PVA hydrogel was prepared according to a previous study [19]. The PVA standard solution was prepared by dissolving PVA (26 g) in distilled water (200 mL) and heated at 80 °C until PVA was completely mixed with the distilled water. Both OPB and MWCNTs were added to 15 g of prepared PVA solution. The temperature of the mixture was controlled about 70 °C with constant stirring. Then, NMBA (0.08 g) and APS (1.25 g) were added and the mixture was constantly stirred until a hydrogel was formed. The as-produced PVA hydrogel was finally washed with acetone, oven dried at 60 °C for 24 h and stored in a desiccator for further use. 

### 2.4. Characterizations of OPB/MWCNTs Reinforced PVA Hydrogel

The internal surface and morphology of as-prepared OPB/MWCNTs reinforced PVA hydrogels were characterized by field emission scanning electron microscope (FESEM, ZeissSupra55 VP, Jena, Germany). The Brunauer–Emmett–Teller (BET) specific surface area (Micrometrics ASAP-2000, Norcross, GA, USA) was employed to measure the surface area and pore diameter of the as-prepared hydrogel [44]. Nitrogen (N_2_) adsorption–desorption is commonly used to investigate the properties by dispersing the sample cell into liquid N_2_. For these measurements, the required amount of hydrogel was measured until 0.2–0.3 g by using sample a tube and then hydrogel was out-gassed at about 130 °C overnight. A mercury porosimeter was used to determine the size and surface area of pores in the grafted composites. Thermal characterization of all hydrogels was studied by using Thermal Gravimetric Analysis (Perkin Elmer, TGA 4000, Waltham, MA, USA) and differential scanning calorimetry (DSC Q200, V24, Columbus, OH, USA). TGA was performed from 20 to 1000 °C at a heating rate of 10 °C/min while DSC analysis was conducted at a heating rate of 5 °C/min from 30 to 500 °C under nitrogen atmosphere [45]. The Fourier transform infrared (FTIR, Shimadzu FTIR-8400S, Billerica, MA, USA) spectrophotometer was used to analyze the functional groups of as-prepared composites in the range of 400–4000^−1^ wavelength. The sample was placed in the sample cell and spectrum was recorded at 20 scans with 4 cm^−1^ resolution. The sample was properly mixed with potassium bromide (KBr) in an agate mortar and pressed into a thin plate form [44]. All prepared PVA hydrogels went through one cycle of heating and cooling process to further determine the exothermic and endothermic characteristics, as well as the crystallinity of the fabricated nanocomposites.

### 2.5. Mechanical Properties of OPB/MWCNTs Reinforced PVA Hydrogel

The universal testing machine (UTM, ASTM D638, Aalborg, Denmark) was used to determine mechanical properties, such as tensile strength, elongation at break and modulus elasticity of the as-prepared PVA hydrogels. The crosshead speed testing was set at 10 mm/min. The compressive strength of water swollen hydrogels to withstand loads was determined by using Equation (1):

CS = (load recorded at break(N)/cross-sectional area (mm^2^). (1)

### 2.6. Batch Adsorption of Pb(II) from an Aqueous Suspension

In typical batch experiments, 2.0 g of as-prepared PVA hydrogel was soaked into a 100 mL of the initially prepared stock solutions. The flasks were placed on an orbital shaker and agitated at a fixed speed of 100 rpm/min for 24 h at room temperature. The samples were collected at different time intervals and filtered through a micron-size filter (0.45 mm). The adsorption quantities of Pb(II) were determined by atomic absorption spectrophotometer (AAS, Agilent 240FS, Santa Clara, CA, USA). Reference experiments were also performed by using MWCNTs. The adsorption capacity of the PVA hydrogel was calculated by using Equation (2):

*q*_e_ = (*C*_0_ − *C*_e_)*V*/*m* × 100 (2)

where, *C*_0_ and *C*_e_ (mg/L) are initial and equilibrium concentrations of contaminants, *V* (L) is referred to the volume and *m* (g) is the mass of sample.

## 3. Results and Discussion

### 3.1. Morphology Analysis

**Figure 1:**
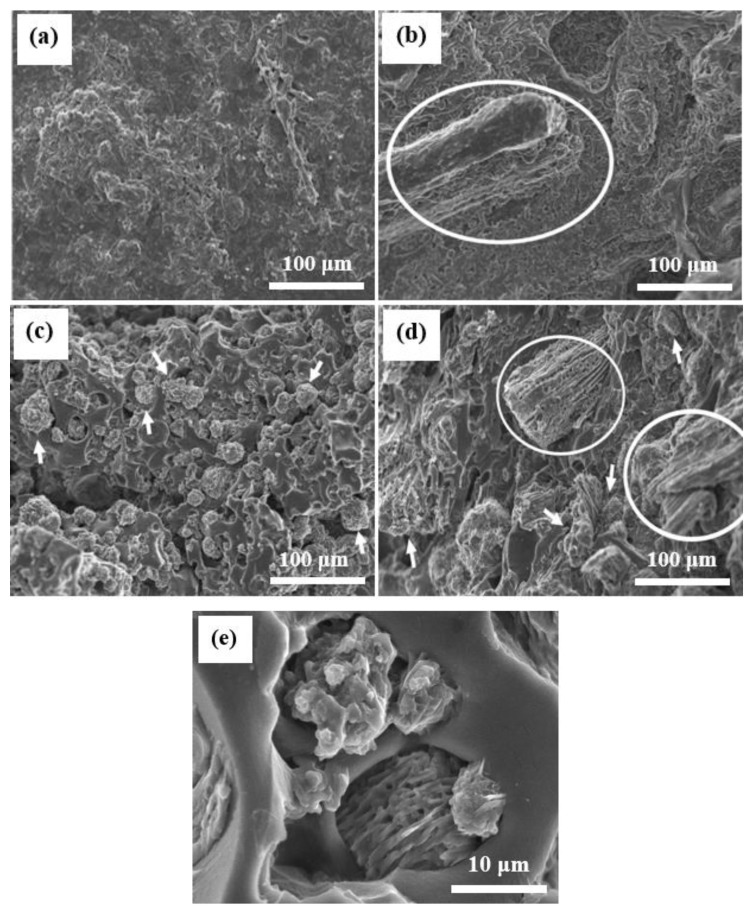
FESEM images of: (**a**) PVA hydrogel, (**b**) oil palm bio-waste (OPB)/PVA hydrogel, (**c**) multiwalled carbon nanotubes (MWCNTs)/PVA hydrogel and (**d**) OPB/PVA/MWCNTs and (**e**) high resolution of OPB/MWCNTs hydrogel, respectively.

Typical FESEM images of non-reinforced PVA, OPB/PVA, MWCNTs/PVA and OPB/PVA/MWCNTs hydrogels are presented in Figure 1. The OPB and PVA blend was employed for the development of hydrogel composites because OPB has poor thermal and mechanical properties, although OPB is expected as a good adsorbent for adsorption due to greater polar side residues than PVA. It can be observed that the morphology of as-prepared reinforced PVA hydrogels was influenced by applying bio-waste and MWCNTs. The small pore size distribution and specific asymmetric pore structures appeared on the non-reinforced PVA hydrogels as shown in Figure 1a; Meanwhile, a white circle revealed a clear presence of OPB over the surface of PVA hydrogel is shown in Figure 1b. The grafting of MWCNTs over the surface of PVA hydrogel is shown in Figure 1c while a clear grafting of unfunctionalized MWCNTs and OPB were formed over the reinforced PVA hydrogels is as shown in Figure 1d. Figure 1e provides more information about the presence of MWCNTs on the surface of OPB. The white circles and arrows indicated the presence of OPB fiber and MWCNTs, respectively. The results illustrated that MWCNTs and OPB were successfully entrenched into the PVA polymer and heavily treated with it. It was observed that the consistency of OPB and MWCNTs with PVA and solid interactions between functional groups of MWCNTs and PVA with OPB was directed to the appropriate hydrogel composites. This showed the strong coordination among OPB, MWCNTs and PVA polymer. The addition of MWCNTs as nanoparticles into the hydrogel composites the increased porosity and pore diameter, which is directly associated to the adsorption performance. Moreover, the surface of reinforced PVA hydrogels was occupied with long strain fiber, producing a fractured surface onto the matrix, which indicated the increment of OPB loading from 0% to 15%. The large nanocarbon agglomerate was formed due to the OPB loading over the fractured surface, which might have a positive influence over the mechanical characteristics of the as-prepared composites [46]. The agglomeration can be avoided through functionalized MWCNTs before the mixing process to obtain an even distribution of nanocarbon in the composite. When the OPB loading increased to 30%, the fractured surface cross-section of the matrix created disoriented protrusions, which might be related to the excellent tensile properties of the reinforced PVA hydrogels. This was due to the stiffening phenomena of fiber loading. The surface cross-section morphology of as-prepared hydrogel composites showed that composite shape was irregular and fractured surface with dense sections. This phenomenon can be explained in terms of improved interfacial interactions between MWCNTs and PVA because of the grafting of MWCNTs onto the OPB particles. In this work, the addition of OPB created a bundling structure, hence, generated an obstacle for water molecules to penetrate the surface matrices.

### 3.2. BET Surface Area Analysis

To further understand the impact of OPB and MWCNTs, BET analysis was performed and the results are shown in Table 2. The addition of MWCNTs and OPB has significantly improved the accessible porosity and pore diameter of OPB/MWCNTs reinforced PVA hydrogel, which was directly associated to the adsorption performance of Pb(II) onto the surface of the PVA hydrogels. These characteristics allowed the water molecules to penetrate the hydrogel and resulted in a high-water uptake capacity. The non-reinforced PVA hydrogel possessed a higher surface area as compared to the reinforced PVA hydrogel. The physical addition of MWCNTs in PVA hydrogel reduced the surface area, which covers the maximum pores of PVA hydrogel. A similar trend was found in other research work [4], whereby porous nanosheet with high porosity and low surface area showed a better Pb(II) adsorption performance as compared to small nanorod, which has a higher surface area. The nano-sized MWCNTs is within the same range of the PVA pore surface size, and thus forms a blockage in the pores, this phenomenon can be seen in Figure 1c,d.

### 3.3. Thermal Analysis

**Figure 2:**
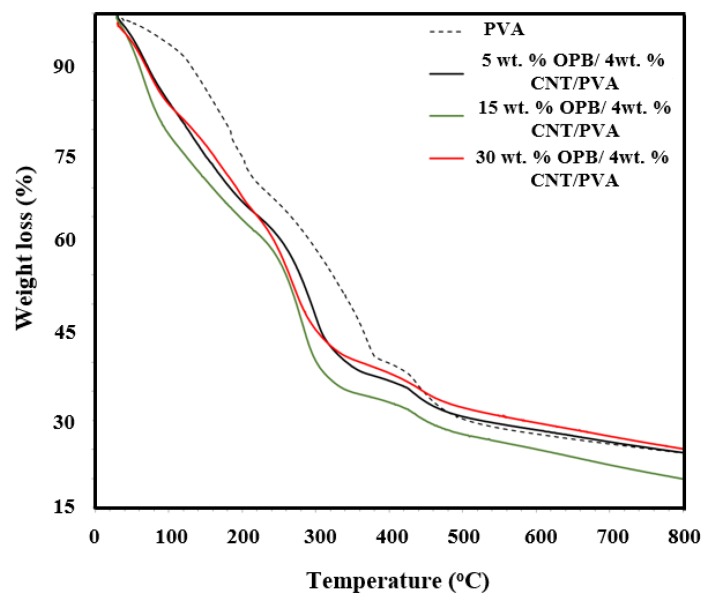
TGA curves of non-reinforced and OPB/MWCNTs reinforced PVA hydrogels.

Figure 2 demonstrates the TGA behavior of non-reinforced PVA hydrogel and OPB/MWCNTs reinforced PVA hydrogels. The TGA results revealed that water vaporization occurred during the heating process. It can be observed that temperature degradation of non-reinforced PVA hydrogel was at 370 °C with a remaining residue of 22.88%. Meanwhile, for the 5 wt % OPB/4 wt % MWCNTs/PVA, temperature degradation was at 298 °C with remained residue of 22.77%, whereas for 15 wt % OPB/4 wt % MWCNTs/PVA was at 300 °C with residue of 17.78%. Temperature degradation of 30 wt % OPB/4 wt % MWCNTs/PVA was slightly delayed to 315 °C with remaining residue of 23.17%. The results showed the ability of grafted adsorbent to withstand high temperature about 300 °C before it started to decompose. As compared to the non-reinforced PVA, the addition of OPB and MWCNTs reduced its thermal stability, and thus showed lower temperature degradation for the reinforced PVA hydrogels. This observation was similar to a previous work [19] in which OPB and MWCNTs attained approximately the same temperature degradation. TGA analysis was divided into two steps. In the first step, water vaporization occurred between 50 and 100 °C while in the second step, the weight loss gradually occurred between 200 and 400 °C due to the degradation of hemicellulose, lignin, and cellulose of OPB [47]. The above conclusions verified more OPB particles, which are in well agreement with FTIR as well as FESEM results.

### 3.4. DSC Analysis

**Figure 3:**
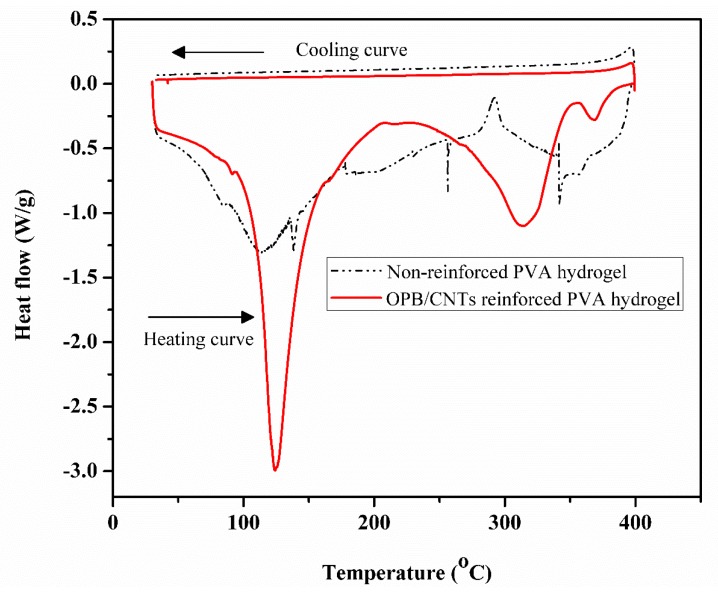
Differential scanning calorimetry (DSC) curves of non-reinforced PVA and OPB/MWCNTs reinforced PVA hydrogels.

Figure 3 shows the DSC behavior of non-reinforced PVA hydrogel and OPB/MWCNTs reinforced hydrogel. The results revealed that one cycle of heating–cooling to demonstrate the endothermic change within a temperature range of 30–400 °C with a slight exothermic difference over the cooling curve of the non-reinforced and OPB/MWCNTs PVA hydrogels. The region between 30 and 50 °C represented the endothermic of water evaporation, which was well correlated with the TGA result. The small region for water evaporation was mainly due to the drying process during the dry hydrogel fabrication. Therefore, very less amount of water was retained in the structure. For the heating curves, non-reinforced PVA hydrogel exhibited a broad endothermic peak, which indicated the melting temperature (*T*_m_) at 110 °C. The loading of OPB and MWCNTs in the structure displayed larger and sharper endothermic curves for reinforced PVA hydrogel. In the reinforced PVA hydrogel, the melting temperature was laid at 15 degrees above the non-reinforced PVA hydrogel. Therefore, the addition of OPB and MWCNTs have strongly improved the capability of the composites to withstand high temperature, and thus increased the melting temperature. The incorporation of MWCNTs and OPB also reduced the enthalpy of fusion (∆*H*). This was because MWCNTs can also act as nucleation agents in the crystallization process that leads to more imperfect crystals, which has been proved in past studies [14]. 

### 3.5. FTIR Analysis

**Figure 4:**
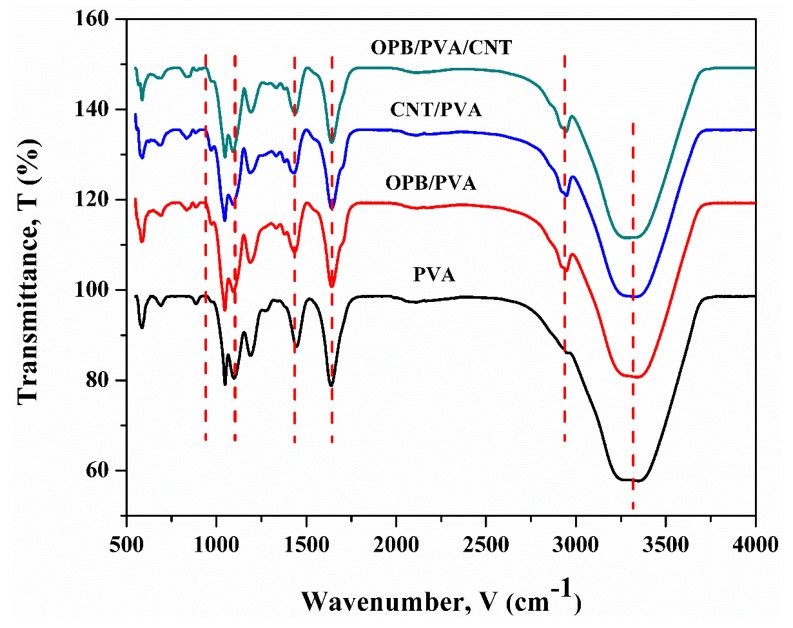
FTIR analysis of PVA, OPB/PVA, OPB/PVA/MWCNTs and OPB/MWCNTs hydrogels, respectively.

FTIR spectra of PVA, OPB/PVA, OPB/MWCNTs and OPB/PVA/MWCNTs hydrogel composites are described in Figure 4. Based on the results, the broad and wide absorption bands were observed between 3550 and 3200 cm^−1^ for all hydrogel composites and represented the stretching of a large number of surface hydroxyl groups (O–H) and adsorption of water from the PVA hydrogel. The peaks around 2900 cm^−1^ were due to the alkyl stretching modes of the PVA backbone. The stretching of C=O was mainly due to the reaction between carboxyl groups of MWCNTs and hydroxyl groups of PVA, which could be observed from the intense peaks obtained at 1668 and 1692 cm^−1^. No significant peak could be observed at this wavelength for PVA and OPB/PVA hydrogels due to the absence of MWCNTs. There were significant peaks at 1401 cm^−1^ for OPB/PVA and OPB/PVA/MWCNTs hydrogel composites due to the C–O bond stretching in the OPB cellulosic fiber. The peak between 1031 cm^−1^ of PVA hydrogels indicated the ester stretch of PVA, and at similar wavelengths, the peaks gradually weakened, indicating that reactions had occurred. The peak at 942 cm^−1^ in PVA hydrogel was associated to the deformation outside the O–H band plan while the peak intensity at 3389 cm^−1^ was related to –OH stretching vibration, which showed good intramolecular as well as intermolecular between H-bond due to the hydrophilicity of the PVA hydrogel. Moreover, the peak intensity at 1095 cm^−1^ was associated to C–O stretching vibration. It was also observed that peaks between 1100 and 1450 cm^−1^ were broadened.

### 3.6. Mechanical Properties

#### 3.6.1. Effect of Multiwalled Carbon Nanotubes (MWCNTs)

**Figure 5:**
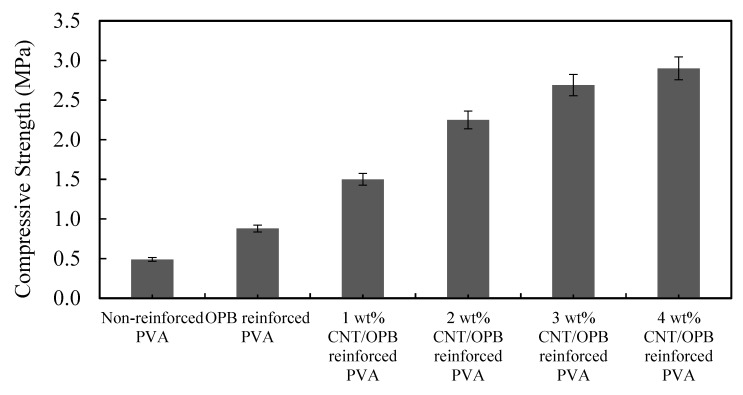
Compressive strength of all water swollen hydrogels at different amount of MWCNTs. Experimental conditions: cross-sectional area of samples = 20 mm^2^, at room temperature.

The ability of the water-swollen hydrogel to retain its shape and endure load was studied by using a compression strength test. It was tested to investigate the properties and loading capacity of fully swollen hydrogels. Compressive strength was defined as the maximum load sustained by a sample in a compressive test divided by the cross-sectional area of specimen. Compressive strength for different MWCNTs loading is shown in Figure 5. According to results, the PVA hydrogel showed minimum mechanical strength of 0.49 MPa, taking a load of 9.8 N for rupture. The addition of OPB was able to improve the mechanical strength of PVA hydrogel to a certain limit. The increment of MWCNTs in all reinforced PVA hydrogels showed excellent improvement in mechanical strength with increased compressive strength from 0.49 to 2.9 MPa. Actually, the addition of MWCNTs into polymer matrices shortened the interlayer distances, which caused the interaction between polymer matrices to strengthen, hence, improved their mechanical characteristics [48,49]. Furthermore, bare MWCNTs was structurally equipped with high aspect ratio, high strength and modulus. This indicated the loading force, which was transferred to the reinforced PVA hydrogels during the compressive test. The maximum compressive value of combined MWCNTs/OPB with 4 wt % showed superior properties to reduce the water absorbency of the PVA hydrogel as compared to others.

#### 3.6.2. Effect of Oil Palm Bio-Waste (OPB)

**Figure 6:**
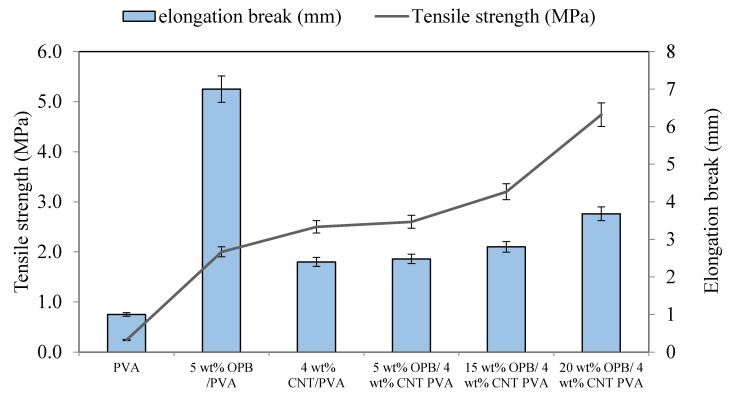
Elongation at break and tensile strength of non-reinforced and PVA hydrogels with 4 wt % MWCNTs at different amount of OPB. Experimental conditions: crosshead speed testing = 10 mm/min, ASTM D638 at room temperature.

Mechanical property is a pivot to estimate the reliability of adsorbents for practical applications. PVA hydrogels with 4 wt % MWCNTs was used with different contents of OPB to investigate the mechanical properties of the hydrogels. Figure 6 shows the elongation at break and tensile strengths of all hydrogels. To further understand the effect of the addition of MWCNTs and OPB, two samples (5 wt % OPB/PVA and 4 wt % MWCNTs/PVA) were synthesized, in which the first PVA hydrogel was synthesized without MWCNTs and the latter without OPB. The results revealed that elongation break had increased tremendously when OPB was introduced into the PVA matrix, however, it was reduced with the addition of MWCNTs. Then, it was gradually increased by increasing the OPB content. It was obvious that the addition of OPB into the pure PVA composite has superbly improved the ability of composite to elongate, which was about 7–9 mm because OPB has strong ability to enhance the ductility of composites. Overall, the OPB/MWCNTs containing PVA hydrogels did not show high elongation break, indicating that the structure possessed high rigidity [50]. For tensile strength, the addition of 4 wt % MWCNTs has greatly improved the tensile strength of PVA hydrogel from 0.2 to 2.2 MPa. This was because the insertion of MWCNTs into the polymer structure shortened the interlayer distances and thus strengthened the interaction between polymer matrices. Furthermore, bare MWCNTs was structurally equipped with high aspect ratio, high strength and modulus. This indicated that the loading force was transferable to nanotubes during stretching. The OPB loading in the composite has improved the tensile strength of the reinforced PVA hydrogels. The highest tensile strength of 4.7 MPa was observed at 20% of OPB while the non-reinforced PVA hydrogel showed the least tensile strength. Therefore, enhancement of mechanical properties was strongly related to electrostatic interaction between filler and matrix. The unique characteristics of MWCNTs, especially its ability to improve the mechanical properties and increase the pore size of PVA-based hydrogel composites make it an excellent adsorbent with perfect mechanical properties showing a good adsorption performance. Moreover, mechanical properties help to form π–π interactions with target pollutants [51].

### 3.7. Batch Adsorption Studies

#### 3.7.1. Effect of Oil Palm Bio-Waste (OPB)

**Figure 7:**
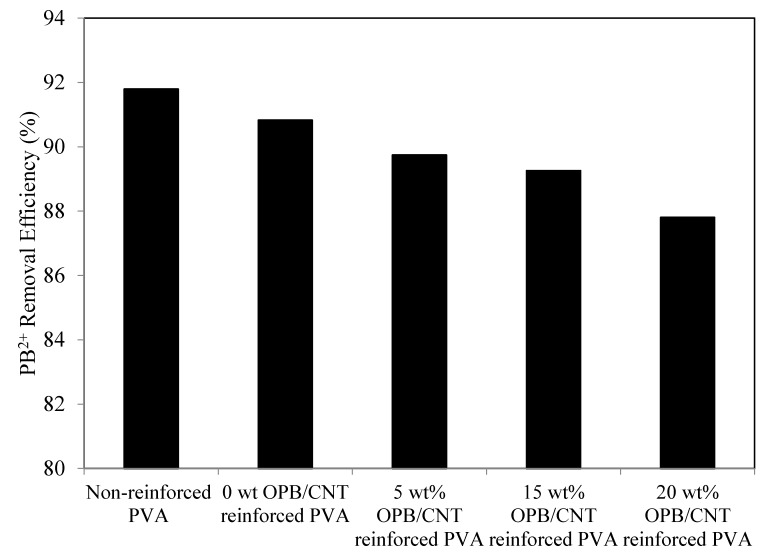
Pb(II) ions removal of non-reinforced and PVA hydrogels with 4 wt % MWCNTs at different amount of OPB. Experimental conditions: adsorbent dose = 0.5 g, Pb(II) concentration = 65 mg/L, pH = 7 and maximum adsorption time = 300 min (1, 3, 5, 10, 20, 30, 60, 120, 180, 240 and 300 min) at room temperature.

The effect of OPB fiber loading over the grafted adsorbents for Pb(II) ions adsorption is shown in Figure 7. The adsorption results revealed that 0 wt % of OPB content had superior Pb(II) ions removal performance (92%), which was gradually decreased with the increased amount of OPB. This showed that the adsorption of Pb(II) ions was closely correlated to the PVA and MWCNTs contents present in the composite. With the persistent quantity of MWCNTs, the increase of OPB addition into the hydrogel composite had minimized a tendency of Pb(II) ions, which were attached over the active sites on a carbon nanotube and caused a decreased trending of adsorption capacity. The reason behind this was that the strong binding of heavy metal ions could enhance over the surface of hydrogel composites through the accumulation of available lone-pair electrons of hydroxyl (–OH) as well as amine (–NH) groups. This was well consistent with the larger specific surface area of pores available into the OPB/CNT reinforced hydrogel, which was supplied more active cites for adsorbing of the Pb(II) ions. Similar results with high capacity of Pb(II) ions adsorption were also reported in previous research work [52].

#### 3.7.2. Effect of Solution pH

**Figure 8:**
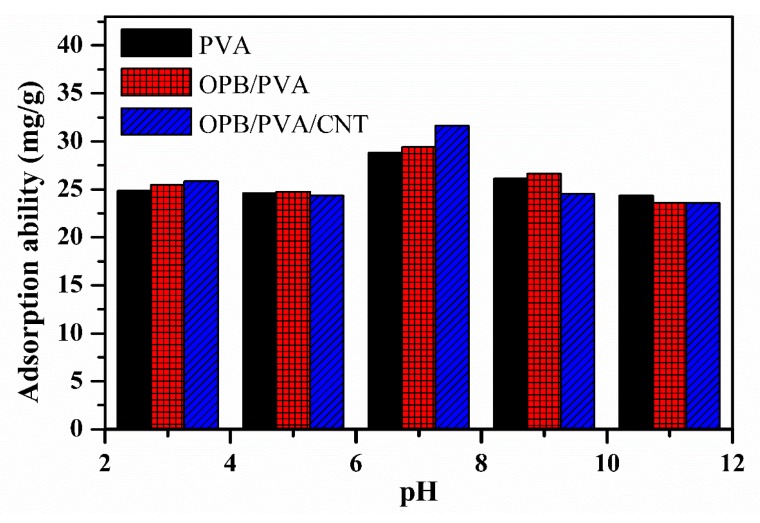
Effect of pH for the adsorption of Pb(II) onto the PVA, OPB/PVA and OPB/PVA/MWCNTs hydrogel adsorbents. Experimental conditions: adsorbent dose = 0.5 g, Pb(II) concentration = 65 mg/L and maximum adsorption time = 300 min (1, 3, 5, 10, 20, 30, 60, 120, 180, 240 and 300 min) at room temperature.

To investigate the effect of initial pH on the hydrogel composites performance, the adsorption of Pb(II) in aqueous solution was measured at different pH values (3–10). The adsorption of Pb(II) was highly dependent on the initial pH of the solution. Figure 8 describes the effect of initial pH on the adsorption of Pb(II). It can be seen that by increasing the pH values from 3 to 7, the adsorption capacity was gradually increased by using PVA, OPB/PVA and OPB/MWCNTs/PVA adsorbents while adsorption capacity was observed to be decreased after increasing the pH to 10. The negative charge on the surface of hydrogel composites was gradually increased by increasing the pH values from 3 to 7 and so the surface of hydrogel composites became more negative at pH 7, which increased the adsorption capacity. The highest adsorption capacity was achieved at pH 7. It could be concluded that the optimized initial pH value for the adsorption of Pb(II) solution was at neutral condition by using these hydrogel adsorbents due to the solid electrostatic interaction between hydrogel composites and Pb(II) ions. The lower pH values were not effective for the adsorption of Pb(II) because of the development of H^+^ ions inside the solution to occupy the active sites of the adsorbents and formation of positive charge on the adsorbents surface [26,32]. Consequently, this can reduce the adsorption performance in acidic pH values. Furthermore, increasing the pH values reduced the adsorption capacity, which could be due to the development of Pb(OH)_2_ and Pb(OH)^+^ [25,26]. Similar investigations were reported in previous studies in, which neutral pH value was also confirmed as optimal pH for the adsorption of Pb(II) by using PVA-based composites [20,32,33]. Therefore, the optimized initial pH value of 7 was explainable.

#### 3.7.3. Effect of Contact Time

**Figure 9:**
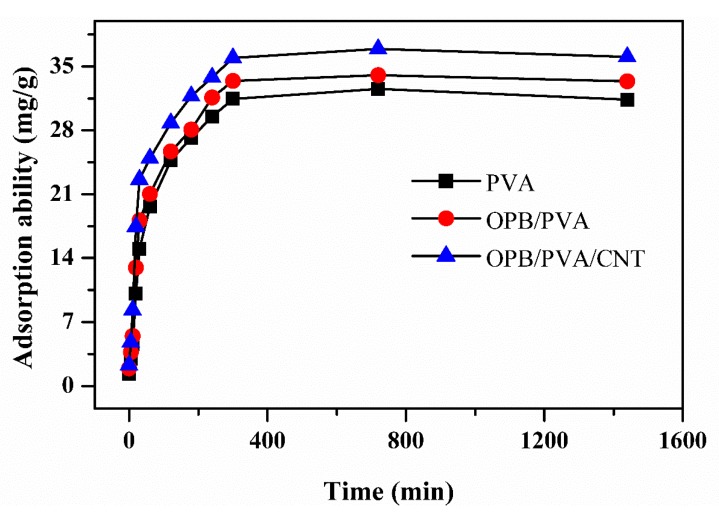
Effect of contact time for the adsorption of Pb(II) onto the PVA, OPB/PVA and OPB/PVA/MWCNTs hydrogel adsorbents. Experimental conditions: adsorbent dose = 0.5 g, Pb(II) concentration = 65 mg/L and pH = 7 at room temperature.

The influence of contact time for the adsorption of Pb(II) onto the PVA, OPB/PVA and OPB/PVA/MWCNTs hydrogel adsorbents is described in Figure 9. The results revealed that the adsorption of Pb(II) strongly depended upon the contact time. It was observed that adsorption capacity of Pb(II) was gradually increased by increasing the contact time to 300 min because more vacant sites were available for the removal of Pb(II). The adsorption capacity was then decreased after increasing the contact time to 1440 min. At this time, the opposition between lead molecules may cause to cover the remaining active sites and reduced the adsorption ability. The enhancement of the adsorption ability from 0 to 300 min was due to the delivered maximum chance for the removal of Pb(II) and the contact time of 1440 min showed least adsorption ability. This proposed that 300 min is the equilibrium time to adsorb the maximum Pb(II) ions onto the surface of hydrogel adsorbents. 

#### 3.7.4. Effect of Pb(II) Concentration

**Figure 10:**
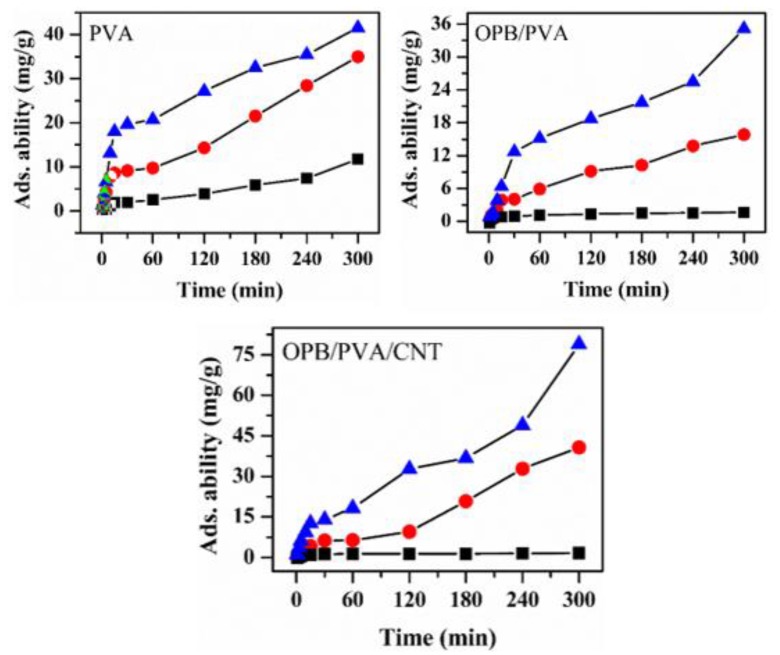
Effect of initial concentrations (65, 150 and 200 mg/L) for the adsorption of Pb(II) onto the PVA, OPB/PVA and OPB/PVA/MWCNTs hydrogel adsorbents. Experimental conditions: adsorbent dose = 0.5 g and pH = 7 and maximum adsorption time = 300 min (1, 3, 5, 10, 20, 30, 60, 120, 180, 240 and 300 min) at room temperature.

The initial effect of Pb(II) concentration was significant to determine the adsorption capability of bio-waste adsorbents. The adsorption of Pb(II) ions between the liquid–solid interfaces is called as metal adsorption process [31]. The influence of initial concentrations of Pb(II) was investigated at different concentrations of 65, 150 and 200 mg/L by using of PVA, OPB/PVA and OPB/PVA/MWCNTs hydrogel adsorbents while other process parameter values were kept constant. The entire Pb(II) concentration range was selected according to the literature [53]. Figure 10 shows the adsorption ability of hydrogel adsorbents at different Pb(II) concentrations. The adsorption of Pb(II) was progressed on the surface of hydrogel adsorbents. The results revealed that the adsorption ability of Pb(II) on the surface of hydrogel adsorbents was a function of the initial concentration of Pb(II). The adsorption capacities of PVA, OPB/PVA and OPB/PVA/MWCNTs hydrogel adsorbents were gradually increased by increasing the concentrations of Pb^2+^ from 65 to 200 mg/L at room temperature. In addition, the adsorption of OPB/PVA/MWCNTs hydrogel adsorbent was higher than PVA and OPB/PVA hydrogels. In general, the higher concentration of Pb(II) rises the driving force and improves the attraction between active sites of the hydrogel adsorbents and Pb(II) ions that produce a concentration gradient between surface of hydrogel adsorbents and bulk solution [29,31].

#### 3.7.5. Adsorption Kinetic Studies

The reaction kinetics analysis provides deep and excellent information about the mechanism and rate of desired adsorption process [4,54]. To understand the reaction kinetic mechanism for the adsorption of Pb(II) on the OPB/MWCNTs reinforced hydrogels, pseudo-first-order (PFO) and pseudo-second-order (PSO) kinetic models were used to investigate the experimental results [55,56]. The adsorption kinetics experiments were performed by using various Pb(II) concentrations of 65, 150 and 200 ppm and pH 7 at room temperature. PFO model is expressed in the following linearized form [57]:

log (*Q*_e_ − *Q*_t_) =log *Q*_e_ − (*K*_1_t/2.303), (3)

where, *K*_1_ (min^−1^) denotes PFO adsorption rate constant while *Q*_e_ (mg/g) and *Q*_t_ (mg/g) express the relative adsorption quantity of Pb(II) at equilibrium condition e and at any time *t* (h), respectively. The graph was plotted against t versus log (*Q*_e_ − *Q*_t_), which achieved the constants values such as *K*_1_ and *Q*_e_ from slopes and intercepts, respectively.

Similarly, the PSO kinetic model was applied to investigate the adsorption mechanism and written in the following linearized form [58]:

*t*/*Q*_t_ = (1/*K*_2_*Q*_e_^2^) + (1/*Q*_e_) × *t* (4)

where, *Q*_e_ (mg/g) and *Q*_t_ (mg/g) express the relative adsorption amounts of Pb(II) adsorbed per unit weight of adsorbent at equilibrium e and at any time *t* (h), respectively. Both *t* (min) and constant *K*_2_ (min^−1^) express the reaction time and PSO chemisorption rate constant, respectively. The graph against *t* versus *t*/*Q*t was plotted and attained the constants from the slope and intercept values.

**Figure 11:**
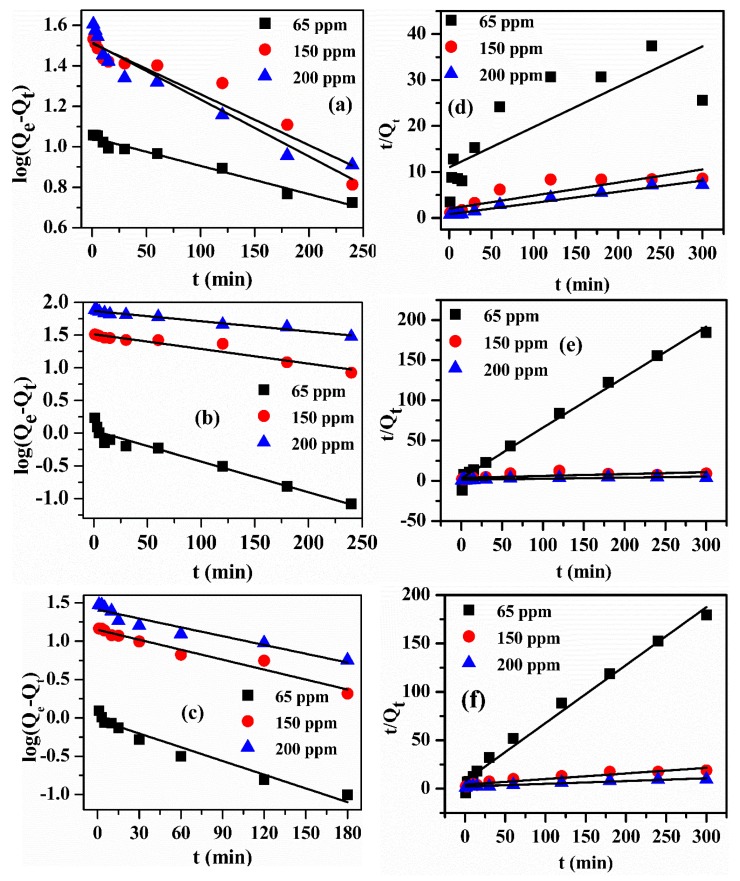
Pseudo-first-order kinetic model of Pb(II) onto the (**a**) PVA, (**b**) OPB/PVA and (**c**) OPB-PVA/MWCNTs while pseudo-second-order kinetic model of Pb(II) onto the (**d**) PVA, (**e**) OPB/PVA and (**f**) OPB-PVA/MWCNTs, respectively at various concentrations (65, 150 and 200 ppm). Experimental conditions: adsorbent dose = 0.5 g, pH = 7 and maximum adsorption time = 300 min (1, 3, 5, 10, 20, 30, 60, 120, 180, 240 and 300 min) at room temperature.

The pseudo-first-order and pseudo-second-order kinetics graphs of PVA, PVA/OPB and OPB-PVA/MWCNTs are described in Figure 11 while, the values of both adsorption kinetics parameters namely *R*_2_, *Q*_e_, cal., *K*_1_ and *K*_2_ are listed in Table 3. The kinetic results revealed that pseudo-second-order kinetic model provided the highest R^2^ values (0.898–0.968, 0.991–0.996 and 0.942–0.998) as compared to the pseudo-first-order kinetic model (0.922–0.974, 0.928–0.977 and 0.916–0.957) by using PVA, OPB/PVA and OPB-PVA/MWCNTs hydrogels, respectively. Moreover, in the case of PSO kinetic model, the experimental and theoretical adsorption values were well correlated with minor difference than the PFO kinetic model. Therefore, PSO kinetic model was proven more suitable to explain the experimental adsorption behavior of Pb(II) over PVA, PVA/OPB and OPB-PVA/MWCNTs adsorbents with more accuracy as compared to the PFO kinetic model. The surface of as-prepared reinforced PVA composite materials was rich in characteristic functional groups such as C=O stretching, which is mainly due to the reaction between carboxyl groups of MWCNTs and hydroxyl groups of PVA and C–O bond stretching that is present in the cellulosic fiber of OPB with Pb(II) chelating ability (chemical adsorption) as well as electrostatic attraction (physical adsorption). Moreover, the adsorption process of Pb(II) over PVA, OPB/PVA and OPB-PVA/MWCNTs adsorbents was considered as a chemical adsorption process, involving valance forces via sharing or exchange of electrons between hydrogel adsorbent and Pb(II) molecules. The chemical adsorption mechanism participated more in the rate of adsorption [45,53]. Based upon this result, the adsorption behavior of Pb(II) was mainly via chemical adsorption. A similar adsorption mechanism was reported in previous studies for the adsorption of heavy metals by using various PVA-based hydrogels as adsorbents [25,27,32]. Moreover, it can be analyzed that the kinetics rate constants (*K*_1_) and (*K*_2_) were gradually decreased with increasing Pb(II) concentrations, which may be due to the much higher competition for the sorption surface active sites at higher Pb(II) concentration. The rate of reaction can be defined in many ways, but the most common way is to measure the change in concentration of a species of the reaction over the adsorption time. If the concentration of the reactant is measured, it will decrease over the time as it is consumed and so the rate of constant could be recorded as negative. On the other hand, if the product is measured then the rate of constant could be taken as positive. In the present study, the values of PFO adsorption rate constants were found to be in negative, which may be due to the measurement of rate for the reactant.

#### 3.7.6. Adsorption Isotherm Studies

The isotherm studies provided useful knowledge regarding mechanism characteristics and adsorbent capability for targeted pollutants through experimental analysis of equilibrium results. It helps to explain the distribution of the sorbate between solid and liquid phases at equilibrium stage [59]. The equilibrium isotherm was analyzed by using different isotherms namely Langmuir, Freundlich and Temkin models using different Pb(II) concentrations of 65, 150 and 200 ppm with pH 7 at room temperature. The Langmuir isotherm was used to described adsorption mechanism through homogeneous equivalent surface site along insignificant interactions. The Langmuir isotherm is expressed in the following linearized form [55,58]:

1/*Q*_e_ = 1/(*K*_L_*q*_m_*C*_e_) + 1/*q*_m_ (5)

where, *q*_m_ (mg/g) denotes the rate constant of Langmuir isotherm, which is associated with the highest adsorption ability. Both *Q*_e_ (mg/g) and *C*_e_ (mg/L) denote the adsorption quantity and equilibrium concentration of Pb(II), respectively. *K*_L_ (L/mg) denotes a constant associated with the adsorption energy. By drawing a graph against 1/*Q*_e_ versus 1/*C*_e_ and estimating the dependent variables namely *q*_m_ and *K*_L_ from the slope and intercept. The Freundlich isotherm is expressed in the following linearized form [60]:

ln *Q*_e_ = *K*_F_ + 1/*n* (ln *C*_e_) (6)

where, *K*_F_ (mg/g) expresses the Freundlich isotherm constant associating to the relative adsorption capacity. *n* is the Freundlich constant and a dimensionless factor, which shows the sorption intensity of adsorption system. The values of Freundlich isotherm constant between 1 and 10 are most favorable for adsorption process, indicating Pb(II) was easily adsorbed by the composite material under experimental conditions. This was ascribed to rich functional groups and hydrogen bonding over the surface of reinforced PVA hydrogel. Moreover, the values of *R*_L_ indicated the shape of the isotherm to be either unfavorable if *R*_L_ > 1, favorable if 0 < *R*_L_ < 1, linear if *R*_L_ = 1 or irreversible if *R*_L_ = 0 [27]. Both *C*_e_ and *Q*_e_ are explained in Langmuir isotherm details. A graph was plotted ln (*Q*_e_) versus ln (*C*_e_) and was helpful to estimate the values of constant parameters including *n* and *K*_F_. Similarly, the Temkin is the most important isotherm for the heterogeneous type adsorption and analyzes the interactions between adsorbent and adsorbate over the surface. The Temkin isotherm is also expressed in the following linearized form [57,61]:

*Q*_e_ = *B*·ln *K*_T_ + *B*·ln *C*_e_ (7)

where, both *K*_T_ (L/g) express the Temkin constant and associating to the adsorption capability while, *B* (J/mol) also indicates the Temkin constant, expressing to the degradation heat. Both constant parameters (*K*_T_ and *B*) were obtained from the intercept and slope of plotting *Q*_e_ against ln (*C*_e_).

**Figure 12:**
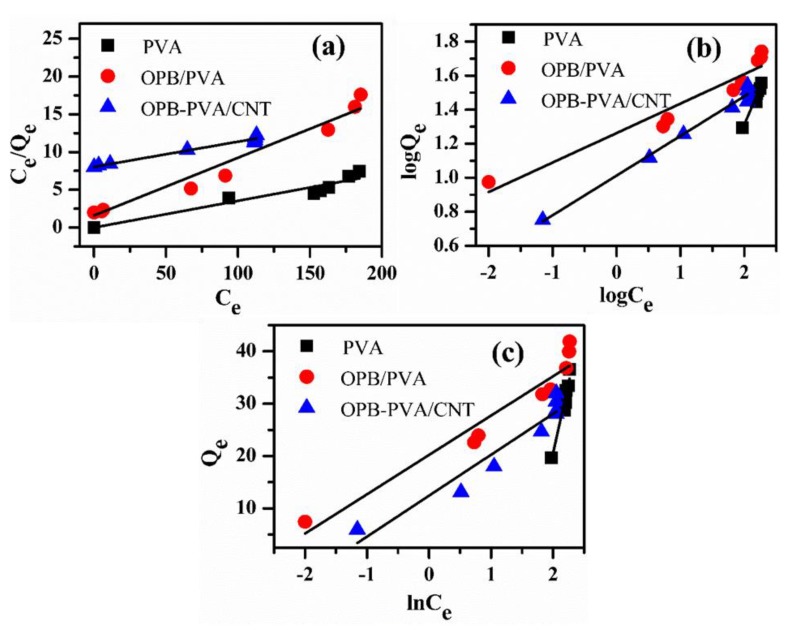
(**a**) Langmuir, (**b**) Freundlich and (**c**) Temkin isotherm models for PVA, OPB/PVA and OPB/PVA/MWCNTs adsorbents for Pb(II) removal at various concentrations (65–200 ppm) and pH 7. Experimental conditions: adsorbent dose = 0.5 g, pH = 7 and maximum adsorption time = 300 min (1, 3, 5, 10, 20, 30, 60, 120, 180, 240 and 300 min) at room temperature.

The three adsorption isotherms are illustrated through linear regressions in Figure 12 while, the values of different isotherm parameters were listed in Table 4. The isotherm results described that the Langmuir isotherm has reported the highest *R*^2^ values (0.973, 0.968 and 0.989) for three adsorbents while Freundlich and Temkin isotherms reported lower *R*^2^ values. The Freundlich isotherm has explained the entire adsorption mechanism with more accuracy suggesting that Pb(II) adsorption over these adsorbents was associated with the monolayer. The value of R_L_ revealed that the isotherm shape and the behavior of the adsorption mechanism [38]. Low *R*_L_ values (less than 1) indicated the strong interaction between Pb(II) and the adsorbent. In this case, OPB/PVA and OPB/PVA/MWCNTs had lower *R*_L_ values showing an excellent interaction with Pb(II). The values of adsorption capacities (*q*_m_) were different for three adsorbents. The adsorbent OPB/PVA/MWCNTs showed the highest adsorption capacity (30.031 mg/g) than the others. Therefore, OPB/PVA/MWCNTs adsorbent had excellent capability to adsorb Pb(II) ions from an aqueous suspension.

#### 3.7.7. Effect of Temperature

**Figure 13:**
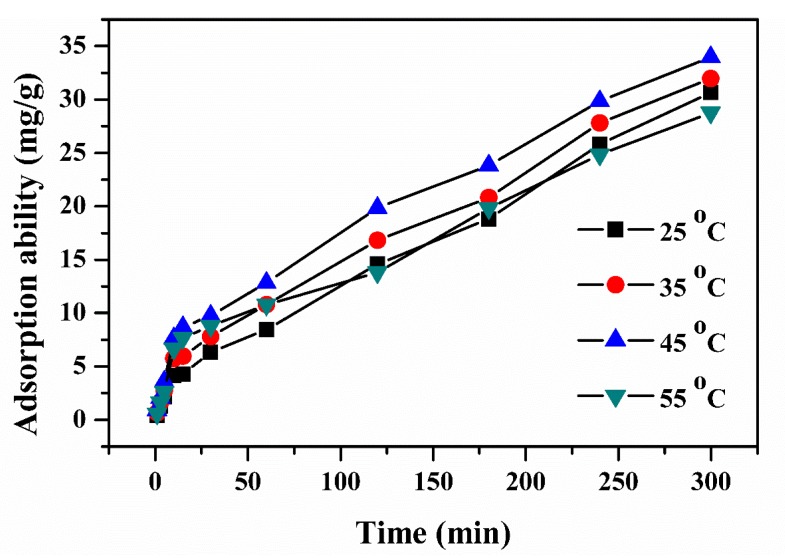
Effect of Pb(II) aqueous solution temperature for maximum adsorption of Pb(II) onto the OPB/PVA/MWCNTs hydrogel.

The influence of temperature of OPB/PVA/MWCNTs hydrogel was described in the range of 25–55 °C and results are illustrated in Figure 13. The results showed that adsorption capacity of Pb(II) was slightly increased by increasing the solution temperature from 25 to 45 °C and then decreased at 55 °C. The adsorption capacity was obtained until 33.986 mg/g at 45 °C than 31.678 mg/g at 25 °C, which means that adsorption capacity of Pb(II) can be progress by rising the temperature to certain range. However, the adsorption capacity curve at 55 °C revealed no further enhancement was related to the adsorption capacity of Pb(II) at 45 °C. This adsorption mechanism of Pb(II) was associated to the intensifying the diffusion speed at relatively higher solution temperature, which made quick and efficient adsorption of Pb(II) over the surface of OPB/PVA/MWCNTs hydrogel and adsorption process was maintained through endothermic process [62]. At higher solution temperature of 55 °C, kinetic energy increased, which resulted in enhanced the adsorption of Pb(II). Moreover, increased adsorption of Pb(II) at relatively higher temperature indicated the fast, feasible and endothermic nature of adsorption process, which was well confirmed by thermodynamics studies. This may suggest that increasing temperature may increase the driving force of Pb(II) over the surface of reinforced PVA hydrogel adsorbent. Such influence was associated to the chemical bonding or reaction that occurred in the adsorption process [63]. Due to the inadequate adsorption sites in OPB/PVA/MWCNTs hydrogel, no further improvement in adsorption capacity of Pb(II) was observed as all adsorption sites were attached with Pb(II) ions and rise in temperature could not show further progress. The same observation has been reported in other study in which tree fern adsorbent was tested for the adsorption of Pb(II) at different temperatures. Although, the increasing temperature of adsorbate solution has not necessarily increase adsorption active sites over the surface of adsorbent but it may generate a new pathway that increase only slightly the rate of adsorption of the adsorbate onto the adsorbent [64]. 

#### 3.7.8. Adsorption Thermodynamics and Activation Energy

The adsorption thermodynamics and activation energy analysis described the nature of adsorption process including spontaneous or non-spontaneous, exothermic or endothermic. The adsorption thermodynamic parameters and activation energy for the adsorption of Pb(II) ions on the OPB/PVA/MWCNTs reinforced hydrogel were investigated at different temperatures such as 25, 35 and 45 °C. The thermodynamic parameters included entropy change (∆*S*°, J/(mol·K)), enthalpy change (∆*H*°, J/(mol·K)), Gibbs free energy (∆*G*°, kJ/mol) and activation energy (*E*_a_, kJ/mol), which are considered as the most important characteristics of adsorption mechanism. The value of Δ*G*° can be estimated by following Equations (8) and (9):

∆*G*° = −*RT*(ln*K*_d_) (8)

∆*G*° = ∆*H*° − *T*∆*S*° (9)

where, *R*, *T*(K) and *K*_d_ illustrates the gas constant (8.314 J/(mol·K)), Pb(II) aqueous solution temperature and coefficient of distribution, respectively. The value of *K*_d_ can be determined by using the following Equation (10):

*K*_d_ = *q*_e_/*C*_e_ (10)

The *q*_e_ and *C*_e_ in mg/L representing the adsorption amount of Pb(II) on the applied hydrogel at equilibrium position and equilibrium concentration of Pb(II), respectively. The values of Δ*G*° lie between 0 and −20 kJ/mol corresponds to physical sorption whereas the values occur between −80 and −400 kJ/mol corresponds to chemical sorption [32]. The values of Δ*H*° and Δ*S*° were calculated by using the following equation by plotting the graph between log *K*_d_ versus 1/*T*.

log *K*_d_ = ∆*S*°/2.303*R* − ∆*H*°/2.303*RT* (11)

**Figure 14:**
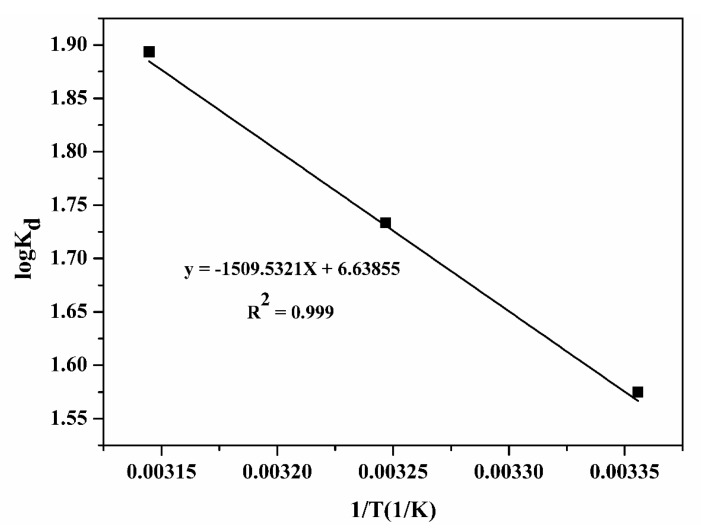
The calculation of adsorption thermodynamic parameters and activation energy by plotting 1/*T*·(1/*K*) versus log *K*_d_.

The Arrhenius equation was helpful to evaluate the activation energies of present adsorption removal process. The activation energy delivers the evidence about the nature of adsorption of Pb(II), whether physical or chemical process. The Arrhenius represents in the following Equation (12):

*K* = *A·*exp(−*E*_a_/*RT*) (12)

where, *K* and *A* are representing gas constant and pre-exponential factor, respectively. The graph between log *K*_d_ and 1/*T* was plotted as shown in Figure 14. The values of enthalpy and entropy change were evaluated by means of slope and intercept. The values of calculated thermodynamic parameters as well as activation energy at different temperatures for the adsorption of Pb(II) by using OPB/PVA/MWCNTs hydrogel are listed in Table 5. The values of *K*_d_ were measured at each temperature and accordingly the value of Δ*G*° was calculated. The thermodynamic results illustrated that the adsorption of Pb(II) on the as-prepared OPB/PVA/MWCNTs hydrogel gradually increased by raising the temperature from 298 to 318 K. By increasing the temperature, the negative values of Δ*G*° were gradually increased from −16.461 to −17.563 kJ/mol indicating that adsorption of Pb(II) on the OPB/PVA/MWCNTs was fast, spontaneous and reasonable process in nature. Moreover, decreasing values of Δ*G*° with rising temperature revealed that adsorption of the Pb(II) was quite satisfactory at higher temperature. A positive value of Δ*H*° was gained, which approved that the adsorption process of Pb(II) over the surface of OPB/PVA/MWCNTs hydrogel was feasible, spontaneous and endothermic in nature. It could be suggested that the endothermic evolution might occur by increasing the Pb(II) solution temperature on the exterior boundary layer and therefore improved the inner pores of applied adsorbent. The positive estimations of Δ*S*° confirmed the increased randomness over the liquid–solid interface during the adsorption process of Pb(II) over the surface of OPB/PVA/MWCNTs hydrogel [31,65]. The results of the activation energy (*E*_a_) confirmed the nature of adsorption process. In the present research, the values of *E*_a_ were found to be 12.551 kJ/mol, which lowered than 40 kJ/mol, indicating the physical adsorption process. The values of *E*_a_ of the chemically controlled process are normally larger than 40 and up to 800 kJ/mol [57]. The reaction controlled by the mixed a mixed mechanism (intermediate-controlled mechanism) showed *E*_a_ in the range from 20 to 35 kJ/mol. The physical controlled process was considered to be readily reversible, equilibrium achieved rapidly and thus energy requirement is small. On the other hand, chemical controlled process was specific and involves relatively stronger forces and thus requirement is larger activation energy [66]. Hence, based upon this results, lower *E*_a_ of reinforced PVA hydrogel attributed to the physical controlled behavior for the adsorption of Pb(II) from aqueous solutions.

#### 3.7.9. Comparison of OPB/PVA/MWCNTs with Other Adsorbents

It is commonly known that adsorption process of Pb(II) was highly dependent upon the temperature than other reaction systems. The activation energies obtained in the present work for the adsorption of Pb(II) (12.551 kJ/mol) with those reported in literature for same pollutant (Pb(II)) through various type of adsorbents were comparable. The activation energies of Pb(II) with different types of adsorbent reported in the range of 4.32–35.528 kJ/mol as listed in the Table 6, which revealed that the adsorption of Pb(II) was mostly physical in nature because the activation energies less than 40 kJ/mol. In this work, activation energy for Pb(II) was comparable to previous works. When comparing the activation energies by using different adsorbents, the significant difference was analyzed for Pb(II), which may also be due to the characteristics of adsorbent applied and experimental conditions and setup. Table 6 shows that by increasing the temperature for the adsorption of Pb(II), the activation energies were gradually decreased.

## 4. Proposed Adsorption Mechanism of Pb(II) onto OPB/MWCNTs Reinforced PVA Hydrogel

**Figure 15:**
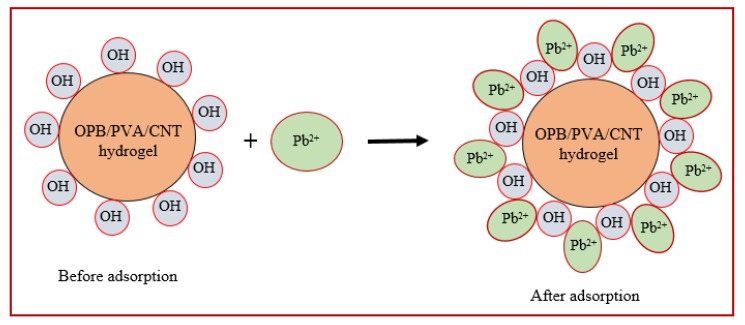
The proposed adsorption mechanism of Pb(II) onto the OPB/MWCNTs reinforced PVA hydrogel composite.

According to adsorption results, a more suitable mechanism way for the adsorption of Pb^2+^ ions onto the OPB/PVA/MWCNTs reinforced hydrogel was proposed. Figure 15 described the proposed removal mechanism of Pb(II) on the OPB/PVA/MWCNTs reinforced hydrogel. Based on the pseudo-second-order kinetic model, the adsorption of Pb(II) on the OPB/PVA/MWCNTs was considered as chemical sorption mechanism [41]. The electrostatic attraction between OPB/PVA/MWCNTs and Pb(II) molecules was highest at pH 7. It can be suggested that these electrostatic attractions between positively charge of Pb(II) ions and negatively charge of OPB/PVA/MWCNTs could successfully promoted the adsorption capacity. The Pb(II) ions may be attached to the oxygen atoms of hydroxyl groups in PVA hydrogel and OPB waste material to enhance the adsorption active sites. These oxygen atoms had great tendency to produce a complex due to the strong attraction of lone pairs electrons. Furthermore, other attraction forces including Van der Waal, hydrogen bonding and hydrophobic may also responsible for the adsorption of Pb(II) on the OPB/PVA/MWCNTs hydrogel. Therefore, electrostatic repulsion forces between OPB/PVA/MWCNTs and Pb(II) caused the highest adsorption. Similar adsorption mechanism was proposed for the adsorption of Pb(II) on the chitosan/PVA hydrogel [22].

## 5. Regeneration of OPB/MWCNTs Reinforced PVA Hydrogel

**Figure 16:**
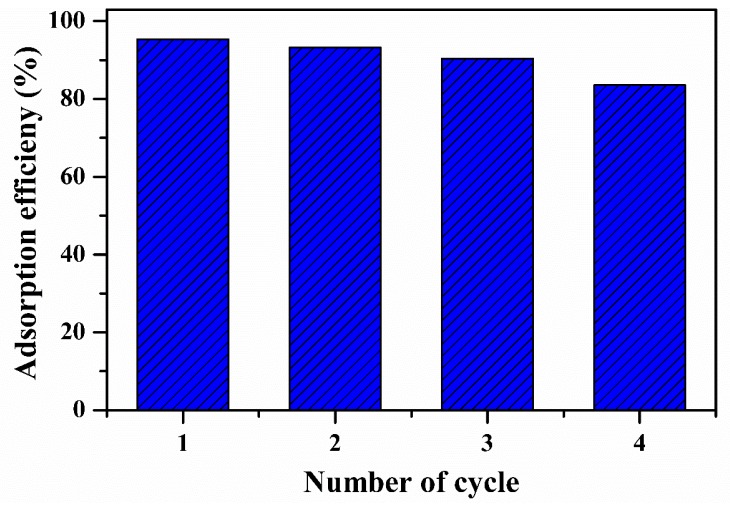
Regeneration of OPB/MWCNTs reinforced PVA hydrogel composite for the adsorption efficiency of Pb(II) at room temperature.

A competent and effective adsorbent with not only excellent adsorption efficiency was required for the contaminants but also efficient reusability efficiency was predictable that may reduce the total price of the adsorbents [66,74]. The desorption experiments were performed at room temperature for four cycles and results are shown in Figure 16. The desorption results cleared that the as-prepared OPB/PVA/MWCNTs hydrogel composite still exhibited excellent adsorption efficiency for Pb(II) as evaluation with first cycle, illustrating well stability for the adsorption of Pb(II) aqueous solution. According to above discussion, as-prepared OPB/PVA/MWCNTs hydrogel could be employed as cheap, efficient and stable adsorbent for the adsorption of Pb(II).

## 6. Limitations and Shortcomings

The incorporation of OPB waste with other polymers and nanomaterials should also be investigated, which may reveal better adsorption capacities for the removal of pollutants. The interval chemistry involved in the synthesis of bio-composites has not been investigated that may provide the valuable understandings for development of best possible bio-composites. The adsorption pathway and intermediates formed during the adsorption process are unknown, which can be evaluated by high performance liquid chromatography (HPLC)/gas chromatography-mass spectrometry (GCMS) analysis.

## 7. Conclusions

The goal of present research was to explore the properties of newly developed reinforced PVA hydrogel composites via employing oil palm bio-waste and MWCNTs for the efficient removal of Pb(II) from aqueous solutions. OPB/MWCNTs reinforced PVA hydrogel composites were prepared in the presence of *N*,*N*′-methylenebisacrylamide (NMBA) as a crosslinking agent and ammonium persulfate (APS) as an initiator via simple in-situ polymerization technique. The thermal and mechanical properties of OPB/PVA/MWCNTs hydrogels provide useful information about the dispersion of MWCNTs into the polymer as well as interfacial interaction between filler and matrix. The characteristics of OPB/PVA/MWCNTs hydrogel composites investigated by FESEM, BET surface area, TGA, DSC and FTIR analysis. The characterization results revealed that the morphological, structural as well as chemical properties of as-prepared reinforced PVA hydrogels was influenced by applying bio waste and MWCNTs. FESEM image analysis demonstrated the presence of OPB as long entanglement and agglomeration of MWCNTs into the structure of the PVA hydrogel matrix. The addition of MWCNTs and OPB have significantly improved the accessible porosity and pore diameter of OPB/MWCNTs reinforced PVA hydrogel, which reduced its thermal stability thus showing a lower temperature degradation for reinforced PVA hydrogels as confirmed through TGA and DSC results. The addition of more OPB in the reinforced PVA hydrogel had reduced its ability to adsorb Pb(II) ions. FTIR spectra showed the improved intensity of the peaks owing to functional groups of hydrogel polymer (C–H stretching and C=O vibration), which acts as reactive adsorption sites and enhanced the adsorption of Pb(II) from aqueous suspensions. The as-prepared reinforced PVA hydrogels were used for Pb(II) ion adsorption test. The experimental adsorption results were well explained through the pseudo-second-order kinetic model and higher *R*^2^ up to 0.998 with small difference between experimental and calculated adsorption outcomes. On the other hand, Langmuir adsorption isotherm was found to be more suitable to describe the adsorption mechanism of Pb(II) onto the as-prepared reinforced PVA hydrogels giving the highest adsorption capacity up to 30.031 mg/g with highest *R*^2^ of 0.989. The various thermodynamic parameters evaluation confirmed that Pb(II) adsorption is fast, spontaneous and endothermic process while lower activation energy (12.551 kJ/mol) revealed the physical adsorption of Pb(II) onto the as-prepared PVA hydrogel. The present outcomes confirm that as-developed hydrogels as novel adsorbents could be used for wastewater treatment application due to improve mechanical, thermal and adsorption properties.

## Figures and Tables

**Table 1:** Different types of polyvinyl alcohol (PVA) hydrogel loaded composites as adsorbents used for the adsorption of Pb(II).

Hydrogel Composite	Solutions	Adsorption Capacity	Ref.
Chitosan/PVA beads	Chitosan/PVA mixed ratio = 75:25–25:75, glutaraldehyde = 5% (*v*/*v*), acetic acid = 1% (wt %), deionized water = 552 g	9.48 mg/g	[25]
Chitosan/PVA blend nanofiber membrane	Chitosan, distilled water, PVA, acetic acid = 2% (wt %)	266.12 mg/g	[26]
Cellulose/chitosan/PVA nanofibrous films	Microcrystalline cellulose = 40 g, water = 4.7 g, H_2_SO_4_ (98 wt %) = 65.3 g, chitosan:acetic acid = 90:10 (vol %), chitosan:PVA = 60:40 (vol %)	323.49 mg/g	[20]
Chitosan/PVA beads	Chitosan flaks = 4.26 g, dilute acetic acid = 2% (*w*/*w*), PVA = 8.51 g, deionized water = 100 mL	-	[22]
Chitosan/PVA thin membrane	Chitosan powder, acetic acid = 1% (*v*/*v*), PVA sol. = 10 wt %, nanodiamonds = 0–1.5 wt %	121.3 mg/g	[29]
Chitosan/PVA talc composite	PVA = 8 wt %, Chitosan = 7 wt %, distilled water, chitosan:acetic acid (concentrated) = 50:50, talc = 1 wt %	88%	[27]
Chitosan/PVA	PVA = appropriate amount, sodium alginate = 1.3 g, CaCO_3_ powder, distilled water = 150 mL, chitosan = certain amount, CaCl_2_-saturated boric acid sol. = 3%	166.44 mg/g	[40]
Chitosan/MWCNTs/PVA hydrogel membrane	Chitosan sol. = 2 wt %, acetic acid sol. = 2 wt %, PVA sol. = 2 wt %	-	[28]
Xanthate-modified with Fe_3_O_4_-based chitosan/PVA hydrogel	Chitosan = 6 g, aqueous acetic acid = 150 mL (2% *v*/*v*), PVA = 6 g, deionized water = 150 mL, Fe_3_O_4_ = 6 g,	97.8%	[30]
Fe_3_O_4_/PVA/spent coffee ground	Spent coffee ground = 100 mesh screen, FeCl_3_/Na_2_SO_3_ = mixed sol., Fe_3_O_4_ particles, PVA sol. = 2 wt % (*w*/*v*), spent coffee ground:Fe_3_O_4_ = 1:1–6:1 (wt %)	0.275 mmol/g	[34]
Chitosan oligosaccharide-g-maleic anhydride/PVA/ silk fibroin composite	Silk fibroin = 0.25 g, 0.5 wt % Na_2_CO_3_, ceric ammonium nitrate = 0.5 g, 1 N HNO_3_ = 10 mL, PVA = 1 mL, chitosan oligosaccharide = 5 g, cocoons, maleic anhydride = 2.5 g, distilled water = 30 mL	16.412 mg/g	[31]
PVA/α-manganese dioxide composite	MnSO_4_.H_2_O = 200 mg, KMnO_4_ = 500 mg, deionized water = 10 mL, ethanol = 9 mL, PVA = 300 mg, H_2_SO_4_ = 1 mL	88.7%	[32]
Graphene oxide/PVA nano-composite hydrogel	Graphene oxide = 0.5 g, deionized water = 100 mL, ethylenediamine-triacetic acid sodium = appropriate amount	67%	[41]
PVA/MWCNTs	MWCNTs = 0.2 g, PVA solution = 500 mL, glutaraldehyde = 10 mL (2.5 %), HCl = 1%	86%	[33]
PVA/graphene oxide-sodium alginate nanocomposite hydrogel	Graphene oxide = 1–5 g, deionized water = 100 mL, sodium alginate = 5 g, PVA = 1–5 g, mixed solution of boric acid and CaCl_2_	279.43 mg/g	[35]
Algal-based sorbent	Polyethyleneimine (PEI) = 6 g, water = 200 mL, glutaraldehyde (50% *w*/*w*) = 6 mL. Alginate/PEI beads: sodium alginate (4% *w*/*w*) = 50 g, PEI derivative powder = 2 g, calcium carbonate sol. (10% *w*/*w*) = 2 g, water = 46 g. Alginate/*Fucus*/PEI beads: *Fucus vesiculosus* alga = 10 g, sodium carbonate = 2 g, water = 288 g, PEI derivative powder = 2 g, sodium alginate = 1 g, calcium carbonate (10 % *w*/*w*) = 2 g, water = 35 g	1.09 mmol/g	[42]
Torrefied biomass	CENTORRE oven = i.d: ϕ 1.82 m, hearth height: 0.74 m including rotatory axis i.d: ϕ 0.42 m, flow rate of biomass ≥ 12 kg/hr, torrefaction conditions = 250 and 280 °C for 75 and 60 min, respectively	30.0 mg/g	[43]
Oil palm bio-waste/MWCNTs/PVA composite hydrogel	PVA = 26 g, distilled water = 200 mL, OPB = 5–20 wt %, MWCNTs = 1–4 wt %, NMBA = 0.08 g, APS = 1.25 g, washed with acetone	30.031 mg/g	This study

**Table 2:** Pore characteristics of hydrogels.

Hydrogels	Accessible Porosity (%)	Total Surface Area (m^2^/g)	Pore Diameter (nm)
PVA	0.07	12.49	8.45
MWCNTs/PVA		2.816	31.11
5 wt % OPB/4 wt % MWCNTs/PVA		2.861	31.54
15 wt % OPB/4 wt % MWCNTs/PVA		2.352	39.75
30 wt % OPB/4 wt % MWCNTs/PVA	7.53	3.382	110.98

**Table 3:** Adsorption kinetic parameters obtained from pseudo-first-order and pseudo-second-order kinetic models using PVA, OPB/PVA and OPB/PVA/MWCNTs adsorbents.

Adsorbents	Experimental		PFO Kinetic Model			PSO Kinetic Model		
	Conc. (ppm)	*Q*_e, exp._ (mg/g)	*Q*_e, cal._ (mg/g)	*K*_1_ × 10^−3^ (min^−1^)	*R* ^2^	*Q*_e, cal._ (mg/g)	*K*_1_ × 10^−4^ (min^−1^)	*R* ^2^
PVA	65	11.121	11.714	−1.401	0.974	11.401	6.981	0.898
	150	33.175	34.969	−2.511	0.922	33.101	4.012	0.868
	200	41.221	41.554	−2.812	0.928	41.011	7.161	0.964
OPB/PVA	65	1.626	1.091	−10.821	0.942	1.591	1252.5	0.991
	150	32.938	32.616	−5.182	0.928	32.735	1.441	0.989
	200	78.912	73.886	−3.591	0.977	78.627	1.372	0.996
OPB/PVA/MWCNTs	65	1.672	1.778	−13.587	0.951	1.763	45.101	0.998
	150	15.817	14.067	−9.991	0.957	15.542	7.641	0.962
	200	35.148	25.763	−8.752	0.916	35.214	3.572	0.942

**Table 4:** Isotherm parameters used for the Pb(II) removal over the PVA, OPB/PVA and OPB/PVA/MWCNTs adsorbents.

Isotherms	Parameters	PVA	OPB/PVA	OPB/PVA/MWCNTs
Langmuir	*q*_m_ (mg/g)	18.329	13.021	30.031
	*K*_L_ (L/mg)	3.534	0.791	0.779
	*R* ^2^	0.973	0.968	0.989
Freundlich	*K*_F_ (mg/g)	−0.449	1.263	1.013
	*n*	1.141	5.772	4.291
	*R* ^2^	0.966	0.922	0.987
Temkin	*K*_T_ (L/g)	0.539	1.451	1.876
	*B*	53.476	7.514	7.821
	*R* ^2^	0.944	0.925	0.915

**Table 5:** Thermodynamic parameters and activation energy for adsorption of Pb(II) onto the OPB/PVA/MWCNTs hydrogel.

*T* (K)	Δ*G*° (kJ/mol)	Δ*H*° (kJ/mol)	Δ*S*° (kJ/mol K)	*E*_a_ (kJ/mol)
298	−16.461	28.904	55.193	12.551
308	−17.012			
318	−17.563			

**Table 6:** Activation energies obtained for the adsorption of Pb(II) using different type of adsorbents.

Type of Adsorbents	Temperature Range (K)	Activation Energy (kJ/mol)	Ref.
Activated carbon	305–315	35.528	[67]
Bael leaves	303–323	22.20	[68]
PVA-based nanofiber membrane	298–318	20.29	[69]
Native bentonite	303–328	16.51	[70]
Activated bentonite	303–328	15.62	[70]
MWCNTs/silica nanocomposite	295–335	15.80	[66]
Spent grain	288–318	12.33	[71]
Manganese oxide coated zeolite	288–328	11.90	[63]
Spent coffee ground	288–328	11.84	[72]
Unmodified kaolinite clay	298–323	11.90, 19.0, 5.12	[64]
Phosphate modified kaolinite clay	298–323	5.64, 10.68, 4.32	[64]
*Saccharum bengalense*	283–333	5.054	[73]
OPB/PVA/MWCNTs hydrogel	298–318	12.551	Present study
